# Acute alterations in glucose homeostasis impact coronary microvascular function in patients presenting with ST-segment elevation myocardial infarction

**DOI:** 10.1007/s12471-020-01366-5

**Published:** 2020-01-17

**Authors:** M. A. van Lavieren, M. Bax, V. E. Stegehuis, T. P. van de Hoef, G. W. M. Wijntjens, R. J. de Winter, K. T. Koch, J. P. S. Henriques, M. Meuwissen, K. D. Sjauw, J. J. Piek

**Affiliations:** 1 Amsterdam UMC, Heart Center, Department of Interventional Cardiology, Amsterdam Cardiovascular Sciences, University of Amsterdam, Amsterdam, The Netherlands; 2 Department of Cardiology, Haga Teaching Hospital, The Hague, The Netherlands; 3 Department of Cardiology, Amphia Hospital, Breda, The Netherlands; 4 Heart Center, Medical Center Leeuwarden, Leeuwarden, The Netherlands

**Keywords:** ST-elevation myocardial infarction, microvascular dysfunction, acute glucose intolerance

## Abstract

**Background:**

Microvascular dysfunction in the setting of ST-segment myocardial infarction (STEMI) is thought to be related to stress-related metabolic changes, including acute glucose intolerance. The aim of this study was to assess the relationship between admission glucose levels and microvascular function in non-diabetic STEMI patients.

**Methods:**

92 consecutive patients with a first anterior-wall STEMI treated with primary percutaneous coronary intervention (PPCI) were enrolled. Blood glucose levels were determined immediately prior to PPCI. After successful PPCI, at 1‑week and 6‑month follow-up, Doppler flow was measured in culprit and reference coronary arteries to calculate coronary flow velocity reserve (CFVR), baseline (BMR) and hyperaemic (HMR) microvascular resistance.

**Results:**

The median admission glucose was 8.3 (7.2–9.6) mmol/l respectively 149.4 mg/dl [129.6–172.8] and was significantly associated with peak troponin T (standardised beta coefficient [std beta] = 0.281; *p* = 0.043). Multivariate analysis revealed that increasing glucose levels were significantly associated with a decrease in reference vessel CFVR (std beta = −0.313; *p* = 0.002), dictated by an increase in rest average peak velocity (APV) (std beta = 0.216; *p* = 0.033), due to a decreasing BMR (std beta = −0.225; *p* = 0.038) in the acute setting after PPCI. These associations disappeared at follow-up. These associations were not found for the infarct-related artery.

**Conclusion:**

Elevated admission glucose levels are associated with impaired microvascular function assessed directly after PPCI in first anterior-wall STEMI. This influence of glucose levels is an acute phenomenon and contributes to microvascular dysfunction through alterations in resting flow and baseline microvascular resistance.

## What’s new?

- Major stress-related metabolic changes occur in the first hours after ST-segment elevation myocardial infarction (STEMI), leading to glucose intolerance in some patients
- Elevated glucose levels and glucose intolerance are associated with an increased risk of mortality, heart failure and cardiogenic shock and no-reflow phenomenon in the culprit vessel
- Elevated admission glucose levels in the setting of STEMI are associated with impaired microvascular function in non-culprit vessels at baseline
- Larger STEMI’s with higher Troponin T levels are associated with higher glucose levels, which may be associated with microvascular dysfunction in non-culprit vessels

## Introduction

It is well recognised that even after rapid and successful revascularisation of ST-segment elevation myocardial infarction (STEMI), myocardial tissue perfusion remains compromised in 30–40% of patients despite restored epicardial patency [1, 2]. This phenomenon is attributed to microvascular dysfunction in the setting of acute STEMI [3], which is observed in both the perfusion territory of the culprit artery, and in non-ischaemic regions remote from the infarcted myocardial tissue [4]. Whereas culprit vessel flow abnormalities have been ascribed to numerous pathophysiological mechanisms, it has partly been ascribed to metabolic consequences of the acute ischemic event [5, 6].

Major stress-related metabolic changes occur during the early hours of STEMI, which include the release of stress hormones such as noradrenaline and cortisol, increased concentration of free fatty acids, and the occurrence of glucose intolerance [7]. As a result, elevated glucose levels are frequently observed in (non-diabetic) STEMI patients, which have been associated with an increased risk of in-hospital mortality, congestive heart failure and cardiogenic shock in patients with and without diabetes [8, 9]. Notably, in patients with STEMI, hyperglycaemia is associated with the no-reflow phenomenon in the culprit vessel, postulated to be a proxy of microvascular dysfunction [10]. It suggests that the acute metabolic changes in STEMI may contribute to microvascular dysfunction in this setting through alterations in glucose homeostasis.

The aim of this study was to assess the relationship between admission glucose levels and microvascular function in non-diabetic patients with first anterior-wall STEMI.

## Methods

A total of 100 consecutive patients with a first anterior-wall STEMI treated by primary percutaneous coronary intervention (PPCI) were enrolled. The initial results were reported previously [4, 11]. STEMI was defined as chest pain lasting >30 minutes in the presence of persistent ST-segment elevation in ≥2 precordial leads. PPCI was performed within 6 hours after onset of symptoms according to standard clinical practice. The exclusion criteria were reported previously [4]. The study protocol was approved by the local ethics committee and all patients gave informed consent.

### Cardiac catheterisation and periprocedural measurements

After successful reperfusion, intracoronary blood flow velocity was measured in the infarct-related artery (IRA) and an angiographic normal reference vessel (diameter stenosis <30% on visual estimation) using a 0.014-inch sensor equipped guide wire (Volcano Corp., San Diego, CA). Reference vessel measurements were performed in the left circumflex coronary artery, or the right coronary artery if a stenosis of >30% was present. At 1‑week and 6‑month follow-up, patients underwent repeat angiography with assessment of intracoronary Doppler flow velocity. Hyperaemia was induced by an intracoronary bolus of 20–40 µg adenosine. Before and after PCI, coronary angiography suitable for quantitative coronary angiographic analysis was performed for offline analysis of thrombolysis in myocardial infarction (TIMI) flow and myocardial blush grade. Left ventricular function was evaluated by means of echocardiographic 16-segment Wall Motion Score Index (WMSI) performed immediately before PPCI.

### Haemodynamic data analysis

Coronary flow velocity reserve (CFVR) was calculated as the ratio of hyperaemic average peak flow velocity (hAPV) to baseline average peak velocity (bAPV). In the absence of significant epicardial disease in the reference vessels, microvascular resistance was calculated at baseline and during hyperaemia, respectively the ratio between mean aortic pressure and mean distal flow velocity at baseline (baseline microvascular resistance—BMR), and during hyperaemia (hyperaemic microvascular resistance—HMR). The delta microvascular resistance from resting to hyperaemic conditions (dMR) was determined by calculating the absolute difference between BMR and HMR.

### Statistical analysis

Normality of the data was tested using the Shapiro-Wilk test, and homogeneity of variance was tested with Levene’s test. All continuous variables are presented as mean ± standard deviation or median [25th–75th percentile] according to their normal or non-normal distribution. Categorical variables are presented as counts and percentages. Univariate regression analysis was used to identify variables associated with reference vessel CFVR at the end of the PPCI procedure (*p*_inclusion_ < 0.1), with candidate variables including all baseline, laboratory and procedural covariates as listed in Tab. 1. Subsequent multivariate analysis was performed using a multivariate linear regression model with adjustments for these variables to identify the association of glucose levels with microvascular function parameters, which are presented as standardised coefficients to facilitate comparison. A *p*-value below the two-sided α‑level of 0.05 was considered statistically significant.

**Table 1:** Baseline clinical and procedural characteristics (*n* = 92)

*Demographics*	
Age, y	56 ± 12
Male	74 (80)
*Risk factors*	
Smoking	49 (53)
Hypertension	23 (25)
Family history	39 (42)
Hyperlipidaemia	24 (26)
*Prior medication use*	
β‑Blocker	12 (13)
Calcium antagonist	8 (9)
Angiotensin-converting enzyme inhibitors	5 (5)
Nitrates	4 (4)
Lipid-lowering drugs	7 (8)
Aspirin	11 (12)
*Laboratory assessment at admission*	
CRP, mg/l	1.9 [1.1–5.2]
Glucose, mmol/l	8.3 [7.2–9.6]
Creatinine, µmol/l	70 [60–79]
NT-proBNP after reperfusion, pg/ml	93 [49–242]
Peak troponin T after 24 hours, ng/ml	4.58 [2.47–6.34]
*Procedural characteristics*	
Heart rate, bpm	79 ± 13
Systolic arterial pressure, mm Hg	119 ± 15
WMSI before reperfusion	1.9 ± 0.2
Time to reperfusion, h	2.9 [2.3–3.9]
ST-segment resolution after reperfusion ≥70%	40 (43)
*Angiographic*	
Final TIMI flow grade 3	56 (60)
Final myocardial blush grade 3	37 (40)

Data are presented as mean ± SD, median [25th–75th percentile], or frequency (%)

*CRP* C-reactive protein,* eGFR* estimated glomerular filtration rate,* NT-proBNP* N-terminal pro-brain natriuretic peptide,* TIMI* thrombolysis in myocardial infarction, *WMSI* Wall Motion Score Index

## Results

In total, 92 patients were included in the study (Tab. 1). Median admission glucose was 8.3 mmol/l [7.2–9.6] respectively 149.4 mg/dl [129.6–172.8] and was significantly associated with infarct size (standardised beta coefficient [std beta] = 0.281; *p* = 0.043), as determined by peak troponin T levels. After PPCI, IRA TIMI 3 flow was achieved in 65 patients (70%). Intracoronary physiological measurements obtained in the IRA and reference vessel are presented in Tab. 2.

**Table 2:** Haemodynamic characteristics

*Infarct-related artery at admission (n* *=92)*	
Final IRA CFVR	1.5 [1.3–1.7]
– Baseline APV, cm per second	19 [14–24]
– Hyperaemic APV, cm per second	29 [21–42]
*Infarct-related artery at 1 week (n* *=62)*	
Final IRA CFVR	1.9 [1.6–2.2]
– Baseline APV, cm per second	21 ± 7
– Hyperaemic APV, cm per second	37 [30–44]
*Infarct-related artery at 6 months (n* *=61)*	
Final IRA CFVR	2.8 ± 0.9
– Baseline APV, cm per second	17 ± 7
– Hyperaemic APV, cm per second	48 ± 19
*Reference vessel haemodynamics at admission (n* *=91)*	
Reference CFVR	2.3 [2.0–2.7]
– Baseline APV, cm per second	16 [14–20]
– Hyperaemic APV, cm per second	37 [31–45]
Baseline MR, mm Hg/cm per second	7.2 [6.2–8.8]
Hyperaemic MR, mm Hg/cm per second	3.1 [2.6–3.8]
Delta MR, mm Hg/cm per second	4.0 [3.3–5.4]
*Reference vessel haemodynamics at 1 week (n* *=62)*	
Reference CFVR	2.7 ± 0.5
– Baseline APV, cm per second	17 [13–20]
– Hyperaemic APV, cm per second	44 [35–53]
Baseline MR, mm Hg/cm per second	6.6 [5.4–8.4]
Hyperaemic MR, mm Hg/cm per second	2.5 [2.1–3.0]
Delta MR, mm Hg/cm per second	4.2 [3.4–5.4]
*Reference vessel haemodynamics at 6 months (n* *=61)*	
Reference CFVR	3.4 ± 0.6
– Baseline APV, cm per second	15 [10–21]
– Hyperaemic APV, cm per second	47 [39–60]
Baseline MR, mm Hg/cm per second	8.9 [6.2–11.3]
Hyperaemic MR, mm Hg/cm per second	2.5 [2.0–3.0]
Delta MR, mm Hg/cm per second	6.0 [4.1–8.3]

Values are presented as mean±SD or median (25th–75th percentile)

*APV* average peak flow velocity,* CFVR* coronary flow velocity reserve,* IRA* infarct-related artery,* MR* microvascular resistance

### Association between admission glucose and microvascular function after PPCI

No association was found between admission glucose levels with CFVR_IRA_, as well as bAPV_IRA_ or hAPV_IRA_ measured directly after revascularisation.

**Fig. 1:**
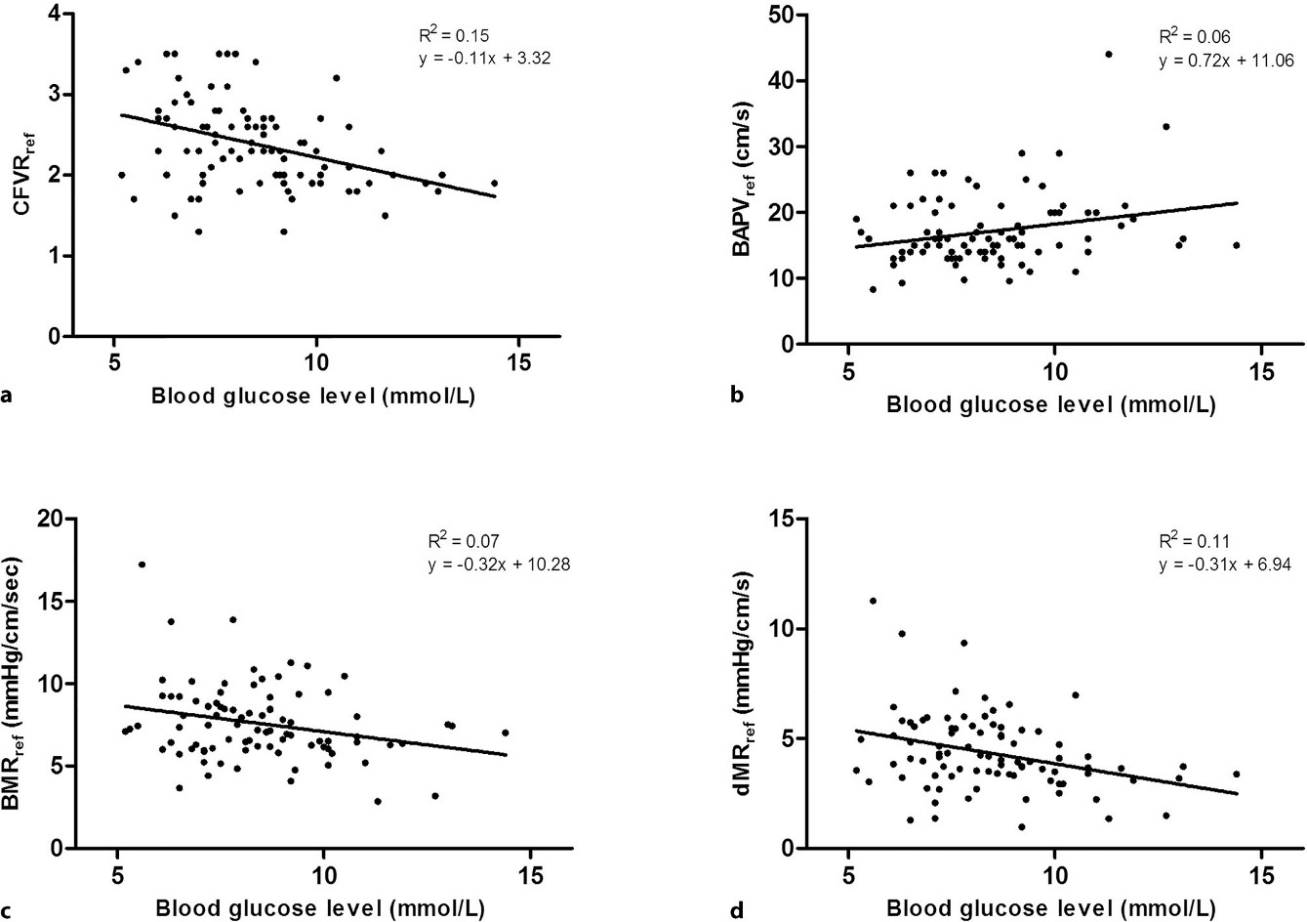
Scatterplots of admission glucose levels with microvascular function in the reference vessel after PPCI. Admission glucose levels were significantly associated with coronary flow velocity reserve (**a**), bAPV (**b**), BMR (**c**) and dMR (**d**) in the reference vessel in the acute setting of STEMI (*PPCI* primary percutaneous coronary intervention, *CFVR*_*ref*_ reference vessel coronary flow velocity reserve, *bAPV*_*ref*_ reference vessel baseline averaged peak velocity, *BMR*_*ref*_ reference vessel baseline microvascular resistance, *dMR*_*ref*_ reference vessel delta microvascular resistance)

CFVR_reference_ decreased significantly with increasing admission glucose levels (std beta = −0.381; *p* < 0.001). In addition, bAPV_reference_ increased significantly with increasing admission glucose levels (std beta = 0.244; *p* = 0.020), and BMR_reference_ decreased with admission glucose levels (std beta = −0.257; *p* = 0.015). Consequently, dMR_reference_ decreased with increasing admission glucose levels (std beta = −0.325; *p* = 0.002) (Fig. 1). hAPV_reference_ as well as HMR_reference_ did not show a significant association with admission glucose levels.

Univariate analysis of all candidate baseline, laboratory and procedural covariates as listed in Tab. 1. Age, heart rate, peak troponin T after 24 hours, WMSI assessed before PPCI, and the use of calcium antagonists were associated with CFVR_reference_. After adjustment for these variables, admission glucose level remained independently associated with CFVR_reference_ (std beta = −0.313; *p* = 0.002), bAPV_reference_ (std beta = 0.216; *p* = 0.033), BMR_reference_ (std beta = −0.225; *p* = 0.038) and dMR_reference_ (std beta = −0.274; *p* = 0.008) (Tab. 3).

**Table 3:** Association between reference CFVR and glucose by univariate and multi-variate analysis at admission, 1‑week and 6‑month follow-up

	At admission (*n* = 92)				At 1‑week follow-up (*n* = 61)		At 6‑month follow-up (*n* = 61)	
	Univariable analysis		Multivariable analysis		Multivariable analysis		Multivariable analysis	
	Std beta	*P* value	Std beta	*P* value	Std beta	*P* value	Std beta	*P* value
*CFVR in reference vessel*								
Glucose	−0.381	<0.001	−0.313	0.002	–	–	–	–
Age	−0.254	0.015	–	–	–	–	–	–
Heart rate	−0.225	0.034	–	–	−0.413	0.002	–	–
Peak troponin T (after 24 h)	−0.469	<0.001	−0.355	0.002	–	–	–	–
WMSI	−0.265	0.014	–	–	–	–	−0.278	0.042
Calcium antagonist	−0.381	<0.001	–	–	–	–	–	–
*Baseline APV in reference vessel*								
Glucose	0.244	0.02	0.216	0.033	–	–	–	–
Age	–	–	–	–	–	–	–	–
Heart rate	–	–	–	–	–	–	–	–
Peak troponin T (after 24 h)	0.241	0.026	–	–	–	–	–	–
WMSI	0.316	0.003	0.266	0.014	–	–	–	–
Calcium antagonist	0.349	0.001	0.385	<0.001	–	–	–	–
*Baseline MR in reference vessel*								
Glucose	−0.257	0.015	−0.225	0.038	–	–	–	–
Age	–	–	–	–	–	–	–	–
Heart rate	−0.262	0.02	−0.229	0.045	−0.269	0.044	–	–
Peak troponin T (after 24 h)	−0.228	0.038	–	–	−0.346	0.022	–	–
WMSI	−0.326	0.003	−0.246	0.035	–	–	–	–
Calcium antagonist	−0.295	0.006	−0.292	0.008	–	–	–	–
*Delta MR in reference vessel*								
Glucose	−0.325	0.002	−0.274	0.008	–	–	–	–
Age	–	–	–	–	–	–	–	–
Heart rate	−0.318	0.004	−0.244	0.023	−0.320	0.015	–	–
Peak troponin T (after 24 h)	−0.376	<0.001	–	–	−0.336	0.022	–	–
WMSI	−0.357	0.001	−0.223	0.041	–	–	–	–
Calcium antagonist	−0.299	0.005	−0.247	0.016	–	–	–	–

*Std beta *standardised beta coefficient, *CFVR *coronary flow velocity reserve,* APV *average peak velocity,* MR *microvascular resistance,* WMS *Wall Motion Score Index

### Association between admission glucose and microvascular function at 1-week and 6-month follow-up

At one week follow-up, intracoronary physiology measurements in the IRA and reference vessel were repeated in 62 patients (Tab. 2). No significant association was found between admission glucose levels and CFVR_IRA_, bAPV_IRA,_ as well as hAPV_IRA_ measured at 1‑week follow-up.

Univariate analysis revealed that admission glucose was significantly associated with CFVR_reference_ (std beta = −0.284; *p* = 0.025), BMR_reference_ (std beta = −0.280; *p* = 0.029), and dMR_reference_ (std beta = −0.295; *p* = 0.021). However, after adjustment for the identified confounders, none of these variables retained a significant association.

At 6‑month follow-up, intracoronary physiology measurements in the IRA and reference vessel were repeated in 61 patients (Tab. 2). Univariate analysis revealed that admission glucose at times of the PPCI was only associated with CFVR_reference_ measured at 6‑month follow-up, although this association was eclipsed after adjusting for the identified confounders. Univariate analysis revealed no association between admission glucose levels, BAPV, hAPV and CFVR at 6‑month follow-up.

## Discussion

We observed that increased admission glucose levels in the acute setting of STEMI are independently associated with alterations in microvascular function, particularly during resting, autoregulated conditions. Increasing glucose levels were associated with progressive impairment of reference vessel CFVR measured directly after PPCI, which resulted from increased bAPV secondary to decreased BMR. At 1‑week and 6‑month follow-up, the existing associations present in the acute setting disappeared, suggesting recovery of coronary autoregulatory function at normalisation of glucose levels.

It has been reported that age, heart rate and infarct size affect myocardial blood flow by influencing myocardial microvascular function [12–15]. Our results confirm this, and add that blood glucose, likely secondary to acute metabolic changes in response to the infarction, plays a distinct role in the pan-myocardial microvascular dysfunction observed in the acute setting of first anterior STEMI.

We found no association between microvascular function and admission glucose levels in the IRA. The influence of admission glucose levels on the parameters of microvascular function was likely eclipsed by other physiological processes that alter microvascular function in the IRA during the acute setting of STEMI.

## Microvascular function following STEMI: novelty of the present findings

Microvascular function assessed by Doppler flow velocity is known to be altered in the setting of STEMI, even in non-ischaemic regions at distance from the infarcted myocardium [4]. We previously reported that microvascular dysfunction in these regions is expressed in an impairment of reference vessel CFVR, which is independently associated with long-term fatal cardiac events [11]. We showed that the acute impairment of reference vessel CFVR in the setting of STEMI originates from a combination of decreased hAPV in the presence of increased HMR, and increased bAPV in the presence of decreased BMR. It has been hypothesised that a combination of mechanical and metabolic alterations due to the acute ischaemic event is responsible for the overall flow impairment at a distance of the infarcted myocardium. The increase in HMR leading to impairment of hyperaemic flow is generally attributed to neurohumoral overactivation[5]. A reduced BMR leading to an increased resting coronary flow may underlie a mechanical as well as a metabolic origin, which is yet to be elucidated. Our present results attribute at least part of the decrease in BMR, and the resulting increase in basal flow velocity, to metabolic changes in the setting of acute STEMI reflected in hyperglycaemia.

## Glucose and insulin mediated microvascular dysfunction

Increased glucose levels are frequently observed in non-diabetic patients presenting with acute myocardial infarction. It reflects the conjoined effects of many interrelated stress mechanisms that influence glucose homeostasis secondary to the acute ischaemic event [7, 16]. Relative insulin resistance is proposed as one of the contributing mechanisms, caused by antagonising effects of stress mediators that impair insulin-regulated glucose uptake [17, 18]. Concomitantly, insulin plays an important role as a mediator in normal myocardial and systemic vascular function [19]. It has been demonstrated to increase myocardial blood flow, acting as a slow vasodilator inducing vasodilation in a time and dose dependent manner [20–22]. In patients with coronary artery disease, intracoronary insulin infusion increases coronary blood flow in the absence of an increase in myocardial oxygen demand [20]. The most important physiological mechanism that contributes to insulin-induced vasodilation is the L‑arginine to nitric oxide pathway in the vascular endothelium [23]. Despite the effects of insulin resistance on glucose uptake and resulting hyperglycaemia, it has been shown that the insulin-induced coronary vasodilation still occurs in obese patients with insulin resistance [24]. Therefore, the association observed between myocardial microvascular function and admission glucose levels might reflect the effect of elevated plasma levels of insulin, secondary to acute relative insulin resistance, on myocardial vascular function. Unfortunately, plasma insulin levels were not measured in the present study and the proposed mechanism of action should be considered hypothesis-generating.

## Concomitant causes of increased baseline flow velocity in STEMI

In addition to the influence of alterations in glucose homeostasis on microvascular function, and in particular bAPV and BMR, other factors may have had a concomitant effect on bAPV. Due to regional myocardial dysfunction, hyperkinesia of remote non-ischaemic myocardium may occur, leading to a predominant increase in bAPV due to an increase in local myocardial oxygen demand [25, 26]. In addition, an increase in left ventricular end-diastolic pressure or stiffening of the myocardium because of hypoxic perfusion, may result in a restriction in myocardial capacitance, leading to an isolated increase in reference vessel bAPV [27, 28]. Nonetheless, the association between admission glucose levels and the bAPV retained significance after adjusting for the identified confounders, including infarct size WMSI which can be considered important predictors for the magnitude of hyperkinesia, left ventricular end-diastolic pressure and hypoxic perfusion.

## Implications for the present study

The present study implies that admission glucose levels are associated with reference vessel microvascular function in the acute setting of STEMI, influencing resting coronary vascular tone and increasing resting flow. Importantly, increased bAPV has previously been documented to be associated with impaired clinical outcomes in both stable coronary artery disease and STEMI [11, 29]. Due to the role of insulin in establishing glucose homeostasis and altering vascular tone, we hypothesise that high insulin levels, secondary to acute insulin resistance, are the mechanism of action responsible for the increase in bAPV. Recovery of this phenomenon at follow-up likely drives recovery of normal coronary autoregulatory function. The fact that larger myocardial infarctions, as determined by troponin T levels, were associated with higher glucose levels, as well as with higher resting flow levels, suggests that the severity of the acute ischaemic event determines the magnitude of metabolic disturbance, and is thereby indirectly related to the magnitude of pan-myocardial microvascular dysfunction.

### Limitations

Accurate assessment of flow velocity depends on the operator’s experience, and, furthermore, on the achievement of maximal vasodilation. The measurements in this study were performed by experienced operators. The amount of adenosine used in this study is considered sufficient [30].

We only assessed reference vessel microvascular resistance in coronary arteries without angiographically significant epicardial narrowing using aortic pressure as a substitute for distal pressure.

In this study, glucose levels were only measured at admission and were not repeated at 1‑week and 6‑month follow-up. This did not allow for exploration of the time course of glucose levels in the period following myocardial infarction. In addition, insulin levels were not determined at any of the time points, resulting in the fact that the hypothesised mechanism could not be further elucidated. Subjects were excluded based on known pre-existing diabetes at the time of admission, however, information on the HbA1C levels was not available to reveal unknown pre-existing impaired glucose homeostasis. Additionally, the study population was relatively small, in particular at 6‑month follow-up, and some statistical analyses may lack statistical significance because of a lack of statistical power.

### Conclusion

Elevated glucose levels at admission for anterior STEMI are associated with impaired microvascular function in myocardial territories remote from the infarction, as assessed by CFVR in reference coronary arteries measured after PPCI. This influence of glucose levels is an acute phenomenon dominantly affecting coronary autoregulation, affecting BMR and bAPV, and contributes to the pan-myocardial microvascular dysfunction observed in acute STEMI.
